# Long-Term Outcomes After Radiofrequency Catheter Ablation of Idiopathic Outflow Tract Premature Ventricular Contractions

**DOI:** 10.3390/medicina62050813

**Published:** 2026-04-24

**Authors:** Sladjana Bozovic-Ogarevic, Zoran Bukumiric, Dejan Kojic, Milovan Bojic, Aleksandra Grbovic, Danijela Tasic, Dragica Dekic, Ljiljana Rankovic-Nicic, Suncica Panic, Marko Filipovic, Zorana Bogicevic, Milan Arsic, Nebojsa Tasic

**Affiliations:** 1 Institute for Cardiovascular Diseases “Dedinje”, 11040 Belgrade, Serbia; kojicdrd@yahoo.com (D.K.); dedinje@ikvbd.com (M.B.); aleksandra_grbovic@yahoo.com (A.G.); dtasic74@yahoo.com (D.T.); gagica81@gmail.com (D.D.); ljiljanabg80@yahoo.com (L.R.-N.); filipovicmarko76@gmail.com (M.F.); zoca005@gmail.com (Z.B.); arsicdr@gmail.com (M.A.); nebtasa@yahoo.com (N.T.); 2 Faculty of Medicine, University of Belgrade, 11000 Belgrade, Serbia; zoran.bukumiric@med.bg.ac.rs; 3 Department of Cardiology, Clinical Hospital Center “Dr Dragisa Misovic”, 11000 Belgrade, Serbia; suncica.panic@gmail.com

**Keywords:** idiopathic premature ventricular contractions, catheter ablation, outflow tract, long-term outcomes, electrophysiology

## Abstract

*Background and Objectives*: Idiopathic ventricular arrhythmias commonly occur in patients without structural heart disease and most often present as premature ventricular contractions (PVCs). Although generally considered benign, a high PVC burden may cause symptoms, reduce quality of life, and lead to reversible PVC-induced cardiomyopathy. This study aimed to evaluate long-term outcomes after radiofrequency catheter ablation of idiopathic outflow tract PVCs. *Materials and Methods*: This single-center retrospective study included 101 patients with idiopathic PVCs who underwent radiofrequency catheter ablation. PVC burden and clinical outcomes were assessed at baseline and during follow-up at 3 months, 12 months, and 5 years. Procedural success, predictors of success, and changes in antiarrhythmic drug therapy were analyzed. *Results*: During follow-up, a marked reduction in PVC burden was observed compared with baseline values. The median PVC burden decreased from 21.89% at baseline to 0.79% at 3 months, 0.23% at 12 months, and 0.09% at the 5-year follow-up after ablation. Acute procedural success was achieved in 88.1% of patients. Long-term success at 5 years was observed in 80.2% of patients. The use of antiarrhythmic drugs decreased during follow-up. Left ventricular ejection fraction remained stable, with no significant difference between baseline and 5-year values. Monomorphic PVC morphology and procedural success at 12 months were identified as independent predictors of long-term success. *Conclusions*: Radiofrequency catheter ablation provides effective and sustained reduction in PVC burden in patients with idiopathic outflow tract PVCs, with high acute success rates, durable long-term outcomes, and reduced reliance on antiarrhythmic drug therapy.

## 1. Introduction

Premature ventricular contractions (PVCs) are among the most common ventricular arrhythmias encountered in clinical practice. They are defined as ectopic impulses originating from the ventricles that result in premature myocardial depolarization [1]. PVCs may occur in both healthy individuals and patients with structural heart disease; however, in the absence of identifiable structural abnormalities, they are classified as idiopathic ventricular arrhythmias [2]. The prevalence of PVCs in the general population is estimated at 1–4% on a standard electrocardiogram, while long-term Holter monitoring detects PVCs in more than 50% of individuals [3].

Although idiopathic PVCs were historically considered benign, accumulating evidence indicates that a high PVC burden may have clinically relevant consequences. Patients with frequent PVCs often report palpitations, fatigue, dizziness, and reduced exercise tolerance, which may significantly affect quality of life [4]. In addition, a high PVC burden has been associated with progressive left ventricular dysfunction and the development of PVC-induced cardiomyopathy (PVC-CMP), a potentially reversible condition following successful elimination of the arrhythmia [5,6,7].

A persistent PVC burden exceeding approximately 10–15% has been associated with early myocardial changes, whereas a burden above 20% is more consistently linked to the development of PVC-induced cardiomyopathy [7,8,9,10]. The mechanisms underlying PVC-CMP are multifactorial and include ventricular mechanical dyssynchrony, prolonged exposure to arrhythmia, and adverse myocardial remodeling [5,7,11].

Idiopathic ventricular arrhythmias most commonly originate from the ventricular outflow tracts and account for approximately 70% of all idiopathic ventricular arrhythmias [12,13,14]. The majority arise from the right ventricular outflow tract (RVOT), while a smaller proportion originate from the left ventricular outflow tract (LVOT) [15]. Idiopathic outflow tract ventricular arrhythmias are primarily driven by cyclic AMP-mediated triggered activity, closely related to delayed afterdepolarizations (DADs) arising from abnormalities in intracellular calcium handling [2,9]. Increased sarcoplasmic reticulum calcium leak and enhanced sympathetic tone, particularly in RVOT arrhythmias, promote DAD formation and facilitate arrhythmia initiation [2]. In addition, repetitive ectopic activity may lead to electromechanical dyssynchrony, contributing to adverse ventricular remodeling over time [7]. While most PVCs originate from focal myocardial sites, involvement of the Purkinje network may further influence arrhythmia characteristics and response to ablation [16].

Radiofrequency catheter ablation (RFCA) is an effective treatment option for symptomatic patients or those with a high PVC burden. Previous studies have reported acute success rates exceeding 85–90%, with sustained reduction in arrhythmic burden in most patients [17,18]. In patients with PVC-induced cardiomyopathy, successful ablation has also been associated with symptom improvement and recovery of left ventricular systolic function [19,20]. From a clinical perspective, catheter ablation is recommended for symptomatic patients with idiopathic PVCs, particularly when antiarrhythmic drug therapy is ineffective, not tolerated, or not desired. In addition, patients with a high PVC burden, especially in the presence of suspected PVC-CMP, may benefit from early intervention to prevent progressive ventricular dysfunction. These considerations highlight the importance of appropriate patient selection and timely referral for ablation.

Although catheter ablation is widely used for the treatment of idiopathic PVCs, long-term outcome data beyond 3–5 years remain limited, particularly in well-defined populations without structural heart disease [13,15,16]. In addition, predictors of sustained procedural success and long-term changes in therapeutic strategies, including antiarrhythmic drug use, are not fully characterized, and clinical outcomes such as mortality and left ventricular function have been inconsistently reported.

Therefore, the present study aimed to evaluate long-term outcomes following radiofrequency catheter ablation of idiopathic PVCs, with a particular focus on procedural efficacy, durability of arrhythmia suppression, changes in antiarrhythmic therapy, and clinical outcomes, as well as to identify predictors of long-term success.

## 2. Materials and Methods

### 2.1. Study Design

This was a retrospective, single-center observational study conducted at the Institute for Cardiovascular Diseases Dedinje, a tertiary referral electrophysiology center. All consecutive patients undergoing RFCA for idiopathic PVCs during the study period were included. Procedures were performed using standardized protocols to ensure consistency across cases. The study design reflects real-world clinical practice in a specialized center.

### 2.2. Ethical Considerations

The study was approved by the Ethics Committee of the Institute for Cardiovascular Diseases “Dedinje” (No. 7548; approved on 13 December 2023). All patients provided written informed consent prior to participation in the study.

### 2.3. Participants

Consecutive adult patients (≥18 years) with frequent symptomatic idiopathic PVCs, refractory to at least one antiarrhythmic drug (AAD) or intolerant to AAD therapy, who were referred for RFCA between November 2018 and December 2023 were included in the study.

During the study period, 146 consecutive patients underwent catheter ablation for PVCs. For the present analysis, only patients with PVCs originating from the ventricular outflow tracts were included. Eligibility criteria required a PVC burden ≥10% on 24 h Holter monitoring or clinically significant symptomatic PVCs, with confirmed outflow tract origin based on activation and electroanatomical mapping.

Patients were excluded if they had structural heart disease, prior ventricular ablation, PVCs originating outside the outflow tracts, or incomplete follow-up data. A total of 45 patients were excluded (structural heart disease, *n* = 15; non-outflow tract origin, *n* = 18; prior ablation, *n* = 7; incomplete data, *n* = 5).

**Figure 1:**
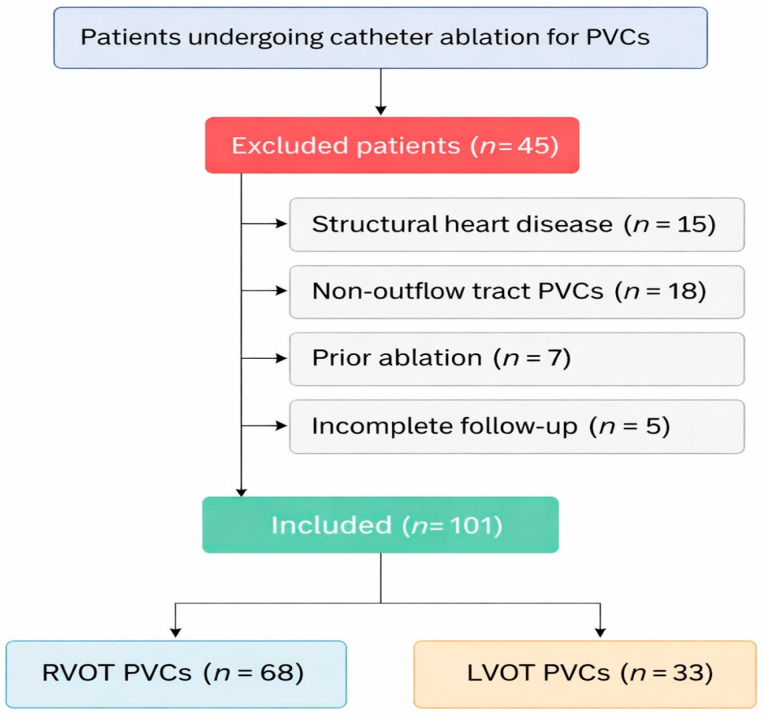
Flowchart of patient selection and study population. Abbreviations: PVC, premature ventricular contractions; RVOT, right ventricular outflow tract; LVOT, left ventricular outflow tract.

The final study cohort consisted of 101 patients. The selection process is illustrated in Figure 1. All patients completed follow-up at 3 months, 12 months, and 5 years.

### 2.4. Data Collection

Clinical, procedural, and follow-up data were collected retrospectively from electronic medical records. Baseline data included demographic characteristics, comorbidities, PVC burden, and echocardiographic parameters. Procedural data included the site of origin (RVOT or LVOT) and acute procedural outcomes. Follow-up data included PVC burden, antiarrhythmic drug use, and clinical outcomes assessed at 3 months, 12 months, and 5 years.

### 2.5. Procedure

Patients were categorized according to the site of ablation into two groups: RVOT and LVOT. All AADs were discontinued at least five half-lives prior to the procedure, except for amiodarone. Procedures were performed under conscious sedation.

In patients with frequent PVCs, localization was determined using activation mapping and pace mapping [16]. The site of origin was defined as the region with the earliest local activation relative to the onset of spontaneous PVCs. In cases of infrequent PVCs, pace mapping was used as the primary technique, with localization based on the highest concordance between spontaneous and pacing-induced PVCs (10 mA/2 ms) on a 12-lead ECG, requiring a match in at least 11 of 12 leads for RFCA to proceed.

If spontaneous PVCs were absent at the beginning of the procedure, programmed ventricular stimulation combined with intravenous isoproterenol administration was used to induce PVCs. Mapping and RFCA were performed under fluoroscopic guidance or using the CARTO electroanatomical mapping system (Biosense Webster, Diamond Bar, CA, USA) with an irrigated-tip ablation catheter (3.5 mm).

For left-sided procedures, intravenous anticoagulation with unfractionated heparin was administered to maintain an activated clotting time (ACT) of 300–350 s. For right-sided procedures, an intravenous bolus of 5000 IU of unfractionated heparin was administered.

### 2.6. Definitions and Outcomes

PVC burden was assessed using 24 h Holter monitoring at baseline (prior to ablation) and during follow-up at 3 months, 12 months, and 5 years. Clinical evaluation and electrocardiographic follow-up visits were performed at the same time points. Transthoracic echocardiography was performed before the procedure and during follow-up to assess LVEF. Clinical outcomes included all-cause mortality and changes in left ventricular systolic function, as assessed by LVEF during follow-up.

Acute procedural success was defined as elimination of the targeted PVC focus, absence of PVCs on ECG monitoring for at least 30 min after the final RFCA application, and non-inducibility of PVCs with or without administration of isoproterenol (0.2–0.6 µg/min) or programmed ventricular stimulation. Clinical success was assessed within 24–48 h based on symptom resolution and absence of PVCs on ECG.

Long-term procedural success was defined as a ≥80% reduction in PVC burden in the absence of antiarrhythmic drug therapy during follow-up. Although no universally standardized definition exists, this threshold has been widely used in prior studies and is supported by expert consensus. It is considered clinically meaningful, as it has been associated with significant symptom improvement and a reduced risk of PVC-CMP [8,17,20].

Procedural complications were systematically recorded and classified as major or minor based on their clinical impact. Major complications were defined as those requiring invasive intervention, prolonged hospitalization, or resulting in permanent sequelae, whereas minor complications were managed conservatively without additional intervention and without long-term consequences.

Complications were further categorized as vascular access-related events, cardiac injury, thromboembolic events, or conduction disturbances. Vascular complications included bleeding, hematoma, pseudoaneurysm, and arteriovenous fistula. Cardiac complications comprised cardiac perforation, pericardial effusion, cardiac tamponade, and myocardial infarction. Thromboembolic events included stroke and systemic embolism, while conduction disturbances included atrioventricular and bundle branch block.

The use of antiarrhythmic medications was recorded before ablation and during follow-up at 3 months, 12 months, and 5 years. The analyzed medications included: (1) beta-blockers; (2) class IC AADs (propafenone, flecainide); (3) class III antiarrhythmic agents (sotalol, amiodarone); and (4) calcium channel blockers (verapamil). The proportion of patients receiving antiarrhythmic therapy was analyzed at each of the specified follow-up time points.

### 2.7. Statistical Methodology

Depending on the type of variables and the distribution normality, data were presented as *n* (%) or mean ± standard deviation. The following statistical tests were used to test the study hypotheses: Student’s *t*-test, Mann–Whitney U test, chi-square test, and Fisher’s exact test.

Logistic regression analysis was used to model the relationship between the dependent variable (5-year procedural success) and potential predictors.

The set of candidate variables was defined based on two criteria: (1) variables that were statistically significant in univariable analyses (*p* < 0.05), and (2) variables identified in the literature as clinically relevant predictors of the outcome. In total, 12 candidate variables were considered for inclusion in the multivariable model.

Given the relatively small number of non-events (*n* = 20) and the imbalance between outcome groups, a parsimonious model was constructed to reduce the risk of overfitting. Variable selection was therefore performed using LASSO (Least Absolute Shrinkage and Selection Operator) regularization, which enables simultaneous variable selection and coefficient shrinkage. The optimal penalty parameter (λ) was determined using cross-validation. As a result, only the most informative predictors were retained in the final model.

Due to multicollinearity, the variable reintervention was excluded from the multivariable model. The final model included two predictors, and with 81 observed events, the events-per-variable (EPV) ratio was 40.5.

Statistical hypotheses were tested at a significance level of α = 0.05, and the results were presented in tables and graphical form. All analyses were performed using IBM SPSS Statistics version 31 (IBM Corporation, Armonk, NY, USA) and the R statistical computing environment (R Core Team, 2025).

## 3. Results

A total of 146 patients underwent catheter ablation for PVCs during the study period. Of these, 101 patients with idiopathic PVCs originating from the ventricular outflow tract were included in the final analysis after applying the predefined exclusion criteria. The patient selection process is presented in Figure 1. The final cohort consisted of 68 patients with RVOT PVCs and 33 patients with LVOT PVCs.

The mean age of the study population was 40.5 ± 8.5 years. A total of 56 patients (55.4%) were female. Hypertension was present in 34 patients (33.7%), hyperlipidemia in 25 (24.8%), and 37 patients (36.6%) were current smokers. Detailed demographic characteristics of the study population are presented in Table 1.

### 3.1. Electrophysiological and Clinical Characteristics

PVCs most commonly originated from the RVOT in 68 patients (67.3%), whereas 33 patients (32.7%) had PVCs originating from the LVOT. The most common presenting symptom was palpitations, reported by 94 patients (93.1%), followed by dyspnea in 60 (59.4%) and fatigue in 54 (53.3%). Dizziness and presyncope, as well as chest pain, were reported less frequently. Detailed electrophysiological and clinical characteristics of the study population are presented in Table 2.

### 3.2. Acute and Long-Term Outcomes

**Figure 2:**
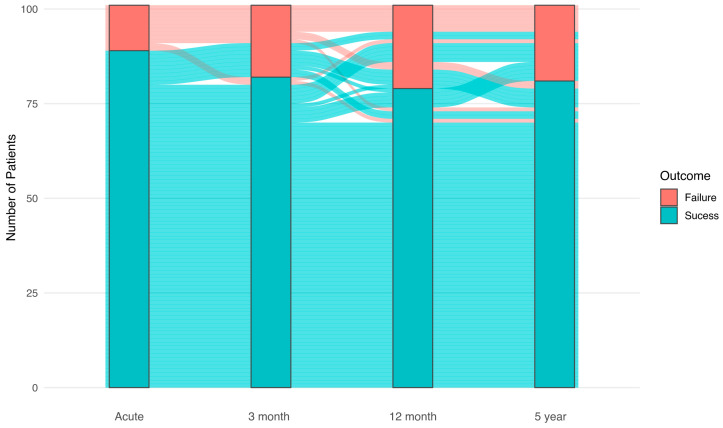
Procedural outcomes of radiofrequency catheter ablation over time. Bars represent the number and proportion of patients with successful and unsuccessful outcomes at each time point (acute, 3 months, 12 months, and 5 years). The flow between bars illustrates transitions in patient outcomes during follow-up.

Acute procedural success was achieved in 89 (88.1%) patients. Procedural success remained high throughout follow-up and was observed in 82 (81.2%) patients at 3 months, 79 (78.2%) at 12 months, and 81 (80.2%) at 5 years (Figure 2), with no significant change over time (*p* = 0.21). These results indicate sustained long-term efficacy of RFCA in reducing PVC burden. No statistically significant difference in long-term success was observed between RVOT and LVOT PVCs (chi-square test, *p* = 0.065), although a trend toward higher success in the RVOT group was noted.

During follow-up, 7 (6.9%) patients required repeat ablation, which was successful in 5 (71.4%). A total of three vascular access complications (3.0%) were recorded, including one hematoma in the RVOT group (1.5%) and two complications (one hematoma and one arteriovenous fistula) in the LVOT group (6.1%). All complications were minor and did not require additional intervention.

No procedure-related mortality or major adverse cardiovascular events occurred during follow-up.

### 3.3. PVC Burden Reduction

During follow-up, a marked reduction in PVC burden was observed compared with baseline values. The median PVC burden decreased from 21.89% at baseline to 0.79% at 3 months, 0.23% at 12 months, and 0.09% at 5-year follow-up after ablation (Table 3).

When stratified according to the site of arrhythmia origin, a substantial reduction in PVC burden was observed in both groups. In the RVOT group, PVC burden decreased from 18.83% at baseline to 0.04% at 5 years. In contrast, patients with LVOT-origin PVCs showed a reduction from 22.88% at baseline to 0.33% at 5 years. Overall, these findings demonstrate a sustained and clinically meaningful long-term reduction in PVC burden following radiofrequency catheter ablation.

### 3.4. Clinical Outcomes

Left ventricular systolic function remained preserved throughout follow-up. The mean LVEF before ablation was 60.28 ± 3.72%, while the mean LVEF at follow-up was 60.69 ± 4.06%. No statistically significant difference was observed between pre-procedural and follow-up values (*p* = 0.447).

### 3.5. Antiarrhythmic Drug Therapy During Follow-Up

Before catheter ablation, the majority of patients were receiving AAD therapy. The most commonly prescribed medications were beta-blockers, used in 75 patients (74.3%), followed by flecainide in 35 patients (34.7%) and propafenone in 30 (29.7%). Sotalol was administered in 20 patients (19.8%), whereas amiodarone and verapamil were used less frequently, in 7 (6.9%) and 3 (3.0%) patients, respectively.

A marked reduction in antiarrhythmic drug use was observed after catheter ablation. At 12-month follow-up, only 17 patients (16.8%) remained on beta-blockers, while the use of other antiarrhythmic drugs markedly decreased (propafenone in 2 patients [2.0%], flecainide in 4 [4.0%], sotalol in 2 [2.0%], and amiodarone in 4 [4.0%]).

This trend persisted during long-term follow-up. At 5 years, beta-blockers remained the most commonly used therapy, used in 15 patients (14.9%), whereas the use of other antiarrhythmic drugs remained low (propafenone in 4 patients [4.0%], flecainide in 5 [5.0%], sotalol in 6 [5.9%], and amiodarone in 2 [2.0%]) (Table 4).

Overall, these findings indicate a substantial and sustained reduction in antiarrhythmic drug therapy following catheter ablation.

### 3.6. Predictors of Long-Term Procedural Success

The final multivariable logistic regression model included two predictors of long-term procedural success, as presented in Table 5, evaluated in 101 patients, of whom 81 experienced the outcome of interest. The overall model was statistically significant (*p* < 0.001) and explained 74% of the variance in the dependent variable (Nagelkerke R^2^). The model demonstrated excellent discrimination, with an area under the receiver operating characteristic curve (AUC) of 0.96 (95% CI 0.91–0.99).

Two variables were identified as independent predictors of long-term procedural success: PVC morphology (OR 75.4, 95% CI 8.4–674.2, *p* < 0.001) and procedural success at 12 months (OR 49.3, 95% CI 5.4–445.9, *p* = 0.001).

Detailed results of the multivariable logistic regression analysis are presented in Table 5.

## 4. Discussion

This study evaluated the efficacy and safety of RFCA in the treatment of idiopathic PVCs, with a particular focus on long-term outcomes. Although PVCs are often reported to be more prevalent in males, the majority of patients in our cohort were female [21]. The study population predominantly consisted of relatively young individuals without structural heart disease, which should be considered when interpreting the external validity of these findings in older patients, those with heart failure, or individuals with more complex ventricular arrhythmias.

Idiopathic PVCs most commonly originate from the ventricular outflow tracts, with approximately 70–80% arising from the RVOT [22]. A comparable distribution was observed in our cohort.

Acute procedural success rates of PVC ablation typically range from 80% to 90% [22,23,24]. In our cohort, the overall acute success rate was 88.1%. PVCs originating from the RVOT are generally associated with higher success rates compared with those arising from the LVOT [25]. Although PVC localization was not a statistically significant predictor of acute success in our study, a numerically lower success rate was observed in PVCs originating from the LVOT, which should be interpreted with caution due to the small sample size.

LVOT arrhythmias may involve more complex anatomy and mapping challenges, including proximity to the aortic cusps and coronary arteries, which can impact procedural strategy and safety considerations. Despite these nuances, contemporary techniques appear to mitigate outcome differences in experienced centers. Procedural failure may be attributed to factors such as intramural or epicardial foci, suboptimal mapping resolution, or limitations in lesion delivery, underscoring the importance of advanced mapping strategies and individualized procedural planning.

Long-term outcomes following PVC ablation are heterogeneous and influenced by multiple factors, including patient characteristics, arrhythmia properties, and the presence of structural heart disease. A meta-analysis by Zang et al. [26] reported success rates ranging from 66% to 90%, reflecting variability across study populations. Higher success rates are typically observed in more selected cohorts, such as patients with frequent RVOT PVCs without structural heart disease [27], whereas lower rates are reported in broader populations, including those with reduced LVEF and more complex arrhythmogenic substrates [28]. Consistent with this, Latchamsetty et al. [17] reported long-term success rates between 71% and 85% in idiopathic PVC populations.

In line with these observations, our study demonstrated sustained efficacy over a 5-year follow-up period following a high rate of acute procedural success, with persistent arrhythmia suppression in the majority of patients.

Beyond arrhythmia control, catheter ablation was associated with a substantial and sustained reduction in antiarrhythmic drug (AAD) use. Evidence from prospective and multicenter studies indicates that catheter ablation achieves high rates of arrhythmia suppression while reducing the need for long-term AAD therapy [4,17]. A large nationwide cohort study further demonstrated significant post-ablation de-escalation of AAD therapy, reflected in both reduced medication use and lower defined daily doses over time [12].

The degree of therapeutic de-escalation varies across patient subgroups. Patients without structural heart disease derive the greatest benefit, whereas those with heart failure often require continued pharmacological therapy despite successful ablation [12]. This distinction has important clinical and economic implications, suggesting that appropriate patient selection may enhance the cost-effectiveness of RFCA. Given the well-recognized limitations of long-term AAD therapy—including proarrhythmic risk, adverse drug effects, and adherence challenges—RFCA represents a strategy that may improve both clinical outcomes and treatment efficiency.

Although randomized data specifically addressing cost-effectiveness in the PVC population are lacking, evidence from other arrhythmia settings suggests that, despite higher upfront procedural costs, catheter ablation may improve quality-adjusted life years (QALYs) and provide a favorable long-term economic profile, particularly when durable rhythm control is achieved [29]. Prospective randomized studies specifically designed to evaluate cost-effectiveness in this population remain warranted.

Regarding safety, the overall complication rate was low (3%) and consistent with previously reported rates of approximately 3–6% [8,17,23]. All observed complications were minor, managed conservatively, and without long-term clinical consequences, and were exclusively related to vascular access. No major complications—defined as those requiring invasive intervention, prolonged hospitalization, or resulting in permanent sequelae—were observed.

A substantial and sustained reduction in PVC burden was observed following catheter ablation, with consistently high rates of long-term arrhythmia suppression over the 5-year follow-up period.

No statistically significant change in LVEF was observed during follow-up. Given the preserved baseline ventricular function in our cohort, LVEF remained stable over time, indicating maintenance rather than recovery of myocardial function. Accordingly, these findings do not support reversal of PVC-induced cardiomyopathy, but instead suggest a preventive effect of ablation in preserving ventricular function.

Reintervention was required in 6.9% of patients, consistent with previously reported rates [17]. Multicenter data indicate that repeat ablation rates range between approximately 5% and 15%, depending on arrhythmia characteristics and follow-up duration [17].

In the present study, multivariable logistic regression identified monomorphic PVC morphology and procedural success at 12 months as independent predictors of long-term procedural success. Monomorphic PVC morphology likely reflects a more localized and stable arrhythmogenic focus, typically of endocardial origin [25,30]. In contrast, polymorphic or variable morphologies may indicate more complex substrates, including intramural or less accessible sites, which are associated with lower ablation efficacy [16]. Furthermore, electrophysiological characteristics such as QRS duration may indirectly reflect the depth of origin, with broader QRS complexes suggesting deeper or intramural foci [30].

The predictive model demonstrated robust performance, explaining a substantial proportion of variance (Nagelkerke R^2^ = 0.74) and showing excellent discriminative ability (AUC 0.96, 95% CI 0.91–0.99). These findings support the model’s utility in identifying patients with a higher likelihood of sustained procedural success.

Procedural success at 12 months emerged as a strong predictor of long-term outcomes, indicating that early arrhythmia suppression is associated with durable elimination of the underlying arrhythmogenic focus. The model should therefore be interpreted as a landmark prediction model rather than a baseline prognostic model.

In the present study, demographic characteristics and comorbidities were not significantly associated with long-term outcomes. Multivariable logistic regression identified monomorphic PVC morphology and procedural success at 12 months as independent predictors of long-term procedural success.

Monomorphic PVC morphology likely reflects a single, well-defined arrhythmogenic focus, facilitating precise activation mapping and effective ablation. In contrast, polymorphic or multifocal ventricular ectopy suggests a more complex arrhythmogenic substrate, which may increase the risk of recurrence [12,15,30]. The association of monomorphic PVC morphology with improved procedural success likely reflects a more localized and stable arrhythmogenic focus, typically of endocardial origin [25,30]. In contrast, polymorphic or variable morphologies may indicate more complex substrates, including intramural or less accessible sites, which are associated with lower ablation efficacy [16]. Furthermore, electrophysiological characteristics such as QRS duration may indirectly reflect the depth of origin, with broader QRS complexes suggesting deeper or intramural foci [30]. Differences between myocardial and potential Purkinje-related PVCs may also contribute to variability in ablation outcomes, further highlighting the importance of detailed substrate characterization [16].

The predictive model demonstrated robust performance, explaining a substantial proportion of variance (Nagelkerke R^2^ = 0.74) and showing excellent discriminative ability (AUC 0.96, 95% CI 0.91–0.99). These findings underscore the model’s utility in identifying patients with a higher likelihood of sustained procedural success. The durability of ablation outcomes likely reflects stable modification of the underlying electrophysiological substrate.

Procedural success at 12 months emerged as a strong predictor of long-term outcomes, indicating that early arrhythmia suppression is associated with durable elimination of the underlying arrhythmogenic focus. The inclusion of 12-month procedural success as a predictor may raise methodological concerns due to its temporal proximity to the outcome and potential for circularity. However, this variable was intentionally retained because it represents a clinically meaningful decision point. In routine clinical practice, patient status at 12 months is used to guide subsequent management, follow-up intensity, and potential additional therapeutic interventions. Therefore, the model should be interpreted as a landmark prediction model rather than a baseline prognostic model.

### 4.1. Clinical Implications

From a clinical perspective, our findings align with current guideline recommendations, supporting a structured approach to patient selection. Catheter ablation should be considered early in symptomatic individuals or in those with a high PVC burden (≥10–15%), not only for symptom control but also for prevention of ventricular dysfunction. Importantly, risk stratification should extend beyond PVC burden alone and incorporate morphological characteristics and early post-procedural response, enabling identification of patients most likely to derive sustained benefit from ablation. Although the sample size was moderate, it is comparable to other single-center studies with long-term follow-up, and the consistency of findings across multiple time points supports the reliability of the results. Furthermore, identification of predictors of long-term procedural success may improve patient selection and support more individualized treatment strategies.

### 4.2. Study Strengths

The present study has several important strengths. First, it provides long-term follow-up data extending to five years after catheter ablation of idiopathic outflow tract PVCs, a duration that remains relatively limited in the existing literature [17,18]. Second, the study includes a well-characterized cohort of patients with strictly defined idiopathic ventricular arrhythmias and comprehensive electrophysiological evaluation. Third, procedural outcomes were systematically assessed using repeated 24 h Holter monitoring, enabling accurate quantification of PVC burden over time [3]. Finally, the analysis of predictors of long-term procedural success provides additional clinically relevant insights that may contribute to improved patient selection and risk stratification.

### 4.3. Study Limitations

This study has several limitations that should be acknowledged. The retrospective observational design inherently carries a risk of selection bias and limits the ability to establish causal relationships between the intervention and the observed outcomes. In addition, referral to a tertiary electrophysiology center may have resulted in a cohort enriched with more symptomatic individuals or those with a higher PVC burden, potentially limiting the representativeness of the general PVC population. Furthermore, the single-center design may restrict external validity, as the findings may reflect specific institutional expertise, procedural strategies, and patient selection practices. The absence of a control group treated with medical therapy precludes direct comparison between RFCA and pharmacological management, while residual confounding due to unmeasured or unavailable factors cannot be entirely excluded. Although electroanatomical mapping systems such as CARTO were already widely adopted at the time of the study, and are currently complemented by other advanced platforms, differences in mapping technologies and operator familiarity may still limit the generalizability of the findings across centers. In addition, the definition of procedural success based on ≥80% PVC reduction, although widely accepted, does not incorporate quantitative symptom assessment or quality-of-life measures, which may limit patient-centered interpretation of outcomes.

Despite the relatively long follow-up duration, the sample size remained moderate, and larger multicenter studies are warranted to confirm these findings. Nevertheless, the consistency of results across multiple follow-up time points, together with objective 24 h Holter monitoring for PVC burden quantification, strengthens the overall validity of the study.

Additional limitations should also be considered. Advanced imaging modalities, such as cardiac magnetic resonance imaging, were not routinely performed, which may limit the strict classification of idiopathic cases despite comprehensive non-invasive evaluation. Furthermore, quality of life and symptom burden were not assessed using validated instruments but were based on clinical evaluation during follow-up. Detailed electrophysiological parameters, including coupling interval variability and circadian distribution of PVCs, were not systematically analyzed due to the retrospective design. Although clinical outcomes such as mortality and left ventricular function were assessed, the study predominantly relies on surrogate endpoints, including PVC burden and antiarrhythmic drug use. Moreover, heart failure was evaluated indirectly based on LVEF, while other hard clinical endpoints, such as hospitalization and composite outcomes, were not systematically analyzed, which may limit the interpretation of long-term prognostic impact.

The inclusion of 12-month procedural success as a predictor may raise methodological concerns due to its temporal proximity to the outcome and potential for circularity. However, this variable was intentionally retained, as it represents a clinically meaningful decision point in routine practice, guiding subsequent management, follow-up intensity, and potential additional therapeutic interventions. Accordingly, the model should be interpreted as a landmark prediction model rather than a baseline prognostic model.

From a statistical perspective, the relatively small sample size, limited number of non-events, and imbalance between outcome groups may affect the stability of the estimates. The observed odds ratios were relatively large and accompanied by wide confidence intervals, suggesting potential imprecision, which may partly reflect both the sample size and the strong association between 12-month procedural success and long-term outcomes. Although LASSO regularization was applied to reduce the risk of overfitting, the number of candidate variables relative to the sample size may still introduce some degree of model uncertainty. In addition, model calibration was not assessed due to the limited number of non-events, which may affect the reliability of calibration estimates. Given that the model includes a clinically relevant but time-dependent predictor, it should be interpreted within a landmark framework. Future studies with larger and more balanced cohorts are warranted to validate these findings.

Further prospective studies integrating detailed clinical, procedural, and cost-related data are needed to better define the optimal timing of intervention and to establish the long-term clinical and economic value of RFCA within contemporary treatment algorithms.

## 5. Conclusions

RFCA represents an effective and safe therapeutic strategy for the treatment of idiopathic premature ventricular contractions, providing high rates of acute success and durable long-term arrhythmia suppression. In addition to significantly reducing PVC burden, catheter ablation is associated with decreased reliance on antiarrhythmic drug therapy and preservation of left ventricular function.

The identification of predictors of long-term procedural success may facilitate improved patient selection and support more individualized treatment strategies.

Overall, these findings support an expanded role of catheter ablation in the management of idiopathic ventricular arrhythmias. Larger prospective studies are needed to confirm these results and to further define the long-term clinical impact of this approach.

## Figures and Tables

**Table 1:** Baseline characteristics according to 5-year procedural outcome.

Characteristic	Total (*n* = 101)	5-Year Success (*n* = 81)	5-Year Failure (*n* = 20)	*p*-Value
Sex, *n* (%)				0.337
Male	45 (44.6%)	38 (46.9%)	7 (35.0%)	
Female	56 (55.4%)	43 (53.1%)	13 (65.0%)	
Age (years), mean ± SD	40.5 ± 8.5	40.7 ± 8.7	39.7 ± 7.8	0.617
Body mass index (kg/m^2^), mean ± SD	25.3 ± 3.3	25.6 ± 3.4	24.1 ± 2.7	0.068
Marital status, *n* (%)				0.699
Married	67 (66.3%)	53 (65.4%)	14 (70.0%)	
Single	34 (33.7%)	28 (34.6%)	6 (30.0%)	
Education level, *n* (%)				0.021
Primary	3 (3.0%)	3 (3.7%)	0 (0.0%)	
Secondary	40 (39.6%)	36 (44.4%)	4 (20.0%)	
Higher	58 (57.4%)	42 (51.9%)	16 (80.0%)	
Employment status, *n* (%)				0.348
Employed	81 (80.2%)	63 (77.8%)	18 (90.0%)	
Unemployed	20 (19.8%)	18 (22.2%)	2 (10.0%)	
Place of residence, *n* (%)				1.000
Urban	90 (89.1%)	72 (88.9%)	18 (90.0%)	
Rural	11 (10.9%)	9 (11.1%)	2 (10.0%)	
Comorbidities and risk factors, *n* (%)				
Hypertension	34 (33.7%)	27 (33.3%)	7 (35.0%)	0.888
Hyperlipidemia	25 (24.8%)	20 (24.7%)	5 (25.0%)	1.000
Diabetes mellitus	8 (7.9%)	5 (6.2%)	3 (15.0%)	0.192
Smoking	37 (36.6%)	27 (33.3%)	10 (50.0%)	0.166
Thyroid dysfunction	14 (13.9%)	11 (13.6%)	3 (15.0%)	1.000

Abbreviations: *n*, number of patients; SD, standard deviation.

**Table 2:** Electrophysiological and clinical characteristics according to 5-year procedural outcome.

Characteristic	Total (*n* = 101)	5-Year Success (*n* = 81)	5-Year Failure (*n* = 20)	*p*-Value
PVC origin, *n* (%)				0.065
RVOT	68 (67.3%)	58 (71.6%)	10 (50.0%)	
LVOT	33 (32.7%)	23 (28.4%)	10 (50.0%)	
PVC morphology, *n* (%)				<0.001
Single morphology	79 (78.2%)	75 (92.6%)	4 (20.0%)	
≥2 PVC morphologies (one dominant)	22 (21.8%)	6 (7.4%)	16 (80.0%)	
Symptoms at presentation, *n* (%)				
Palpitations	94 (93.1%)	74 (91.4%)	20 (100.0%)	0.340
Dyspnea	60 (59.4%)	47 (58.0%)	13 (65.0%)	0.569
Fatigue	54 (53.3%)	42 (51.9%)	12 (60.0%)	0.513
Dizziness and presyncope	6 (5.9%)	5 (6.2%)	1 (5.0%)	1.000
Chest pain	7 (6.9%)	6 (7.4%)	1 (5.0%)	1.000

Abbreviations: PVC, premature ventricular contractions; RVOT, right ventricular outflow tract; LVOT, left ventricular outflow tract.

**Table 3:** PVC burden over time.

Group	*n*	Baseline (%)	3 Months (%)	12 Months (%)	5 Years (%)	*p*-Value
Overall	101	21.89 (8.76–42.99)	0.79 (0.00–24.33)	0.23 (0.00–22.89)	0.09 (0.00–14.34)	<0.001
RVOT	68	18.83 (9.00–42.99)	0.62 (0.00–21.98)	0.16 (0.00–22.89)	0.04 (0.00–13.22)	<0.001
LVOT	33	22.88 (8.76–40.89)	1.23 (0.00–24.33)	0.35 (0.00–21.60)	0.33 (0.00–14.34)	<0.001

Abbreviations: RVOT, right ventricular outflow tract; LVOT, left ventricular outflow tract. Data are presented as median (range). *p*-values were calculated using the Friedman test.

**Table 4:** Antiarrhythmic drug use before and after catheter ablation.

Period	β-Blocker *n* (%)	Propafenone *n* (%)	Flecainide *n* (%)	Sotalol *n* (%)	Amiodarone *n* (%)	Verapamil *n* (%)
Before ablation	75 (74.3%)	30 (29.7%)	35 (34.7%)	20 (19.8%)	7 (6.9%)	3 (3.0%)
12 months	17 (16.8%)	2 (2.0%)	4 (4.0%)	2 (2.0%)	4 (4.0%)	0 (0.0%)
5 years	15 (14.9%)	4 (4.0%)	5 (5.0%)	6 (5.9%)	2 (2.0%)	0 (0.0%)

**Table 5:** Multivariable logistic regression analysis of predictors of long-term procedural success.

Variable	B	OR	95% CI	*p*-Value
PVC morphology	4.323	75.4	8.4–674.2	<0.001
Procedural success at 12 months	3.897	49.3	5.4–445.9	0.001

Abbreviations: B, regression coefficient; OR, odds ratio; CI, confidence interval; PVC, premature ventricular contraction.

## Data Availability

The data supporting the findings of this study are available from the corresponding author upon reasonable request.

## Abbreviations

The following abbreviations are used in this manuscript:

RFCA	radiofrequency catheter ablation
PVCs	premature ventricular contractions
ECG	electrocardiogram
RVOT	right ventricular outflow tract
LVOT	left ventricular outflow tract
PVC-CMP	premature ventricular contraction–induced cardiomyopathy
LVEF	left ventricular ejection fraction
AAD	antiarrhythmic drug
